# Opportunistic Cryptococcal Antigenemia in the HAART Era at HIV Epidemic Settings of Northwest Ethiopia

**DOI:** 10.1155/2020/5017120

**Published:** 2020-09-07

**Authors:** Markos Negash, Tadelo Wondmagegn, Fitsumbrhan Tajebe

**Affiliations:** University of Gondar, College of Medicine and Health Sciences, School of Biomedical and Laboratory Sciences, Department of Immunology and Molecular Biology, Gondar, Ethiopia

## Abstract

**Background:**

*Cryptococcus neoformans* is a frequent opportunistic infection in patients with the acquired immunodeﬁciency syndrome. While the advent of ART reduces the occurrence of cryptococcal meningitis in HIV patients, cryptococcal disease remains a leading cause of morbidity and mortality in the developing world especially in sub-Saharan Africa which is the epicenter of HIV. This study aimed to assess the cryptococcal antigenemia, CD4+ Th cell counts, HIV RNA viral load, and clinical presentations among HIV-positive patients in Northwest Ethiopia.

**Method:**

A total of two hundred (200) HIV-positive patients were recruited for this study. Cryptococcus antigenemia prevalence in plasma samples of HIV‐positive patients was determined by using Antigen lateral ﬂow assay (CrAg‐LFA) also, and CD4+ Th cell counts and HIV‐RNA levels were quantified from blood specimen. Patients' demographic data, clinical manifestation, and concurrent opportunistic infection were recorded.

**Result:**

The sex distributions of study participants were 105(52.5%) male and 94(47.5%) female with an age range of 15–65 (mean 39.42 ± 9) years. All patients had a CD4+ T-cell count <100 cells/*µ*l with the median 54 cells/*μ*l and median HIV-RNA viral load 2.16 × 10^5^ RNA copies/ml (50–3.66 × 10^5^ RNA copies/ml); the prevalence of cryptococcal antigenemia was found to be 4% in HIV-positive patients. More than half and two third of CrAg‐positive patients had a CD4 count <25 cells/*μ*l and HIV viral load >10,000 copies/ml, respectively, as well; Tuberculosis, Candidiasis, and herpes zoster are the most often observed concurrent infections while cryptococcal antigenemia is significantly associated with oral candidiasis (*p* < 0.001).

**Conclusion:**

Although the advent of ART, early diagnosis of cryptococcosis, and application of antifungal interventions, HIV-induced cryptococcal antigenemia positivity in HIV infected individuals is still the countries' big challenge. Thus, stringent follow-up and case management should be considered.

## 1. Background

Cryptococcosis is a potentially life‐threatening systemic disease caused by encapsulated yeast, *Cryptococcus* species [1, 2]. *Cryptococcus neoformans* is the leading cause of meningitis among HIV infected persons with the involvement of the central nervous system and resultant of cryptococcal meningoencephalitis [3, 4]. Although epidemiology is worldwide, morbidity and mortality due to cryptococcal meningitis (CM) vary regionally which is highly influenced by acquired immunodeficiency syndrome (AIDS) [5].

Cryptococcus is common in the environment and most people inhale it, but the fungus usually does not cause serious illness in immunocompetent individuals. Nevertheless, in advanced HIV/AIDS cases, it results in a serious opportunistic infection due to a state of immunecompromisation [6–8]. Following inhalation, *cryptococcus* is recognized by innate receptors of alveolar macrophages though these same immune cells are used by the fungus as an immune evasion system and ensure spreading from the lungs to the brain [9–13].

Worldwide occurrence of cryptococcal infection among HIV patients has been documented in multiple studies [14–18]. Globally, nearly 1 million cases of CM are diagnosed annually, of which the majority are among AIDS patients and accounts 25–30% of HIV/AIDS deaths due to CM [19]. Although the presence of HAART and antifungal interventions for HIV patients has led to the decrement of occurrence, CM is still the principal cause of morbidity if not mortality over *tuberculosis* among HIV-positive patients living in Africa [20–22].

In sub-Saharan Africa where HIV is more widespread and one among three HIV infected individuals is with advanced disease state [23, 24], *C*. *neoformans* is responsible for 42% to 71% of neuromeningitis deaths in HIV infected individuals [25]. In Ethiopia, a known epicenter of HIV, cryptococcal antigenemia is highly prevalent among HIV infected patients with an estimated incidence of 3.4% (Mekelle), 8.4% (Addis Ababa), 10.2% (Oromia), and 11.7% (Bahirdar) [26–29].

The World Health Organization (WHO) has recommendations of cryptococcal antigen (CrAg) test and antifungal therapy for HIV infected patients with CD4+ T-cell count <100 cells/*µ*L [30]. The so-called “targeted screening” of HIV infected patients for CrAg allows early identification of population at high risk and prevention of the establishment of infection, thereby reduction of CM and death.

In Ethiopia, where the burden of HIV is massive, HIV-induced cryptococcal antigenemia is expected to increase. For proper management and control of the infection, determining the disease occurrence has unquestionable importance. Therefore, in this study, we assessed cryptococcal antigenemia on HIV infected individuals at north Gondar, Northwest Ethiopia.

## 2. Methods

### 2.1. Study Design and Settings

A hospital-based cross-sectional study was conducted at the University of Gondar Specialized Referral Hospital ART Clinic from March 2018 to May 2019. The hospital is positioned in the heart of Gondar town, which is located northwestern part of Ethiopia, around 738 km from the capital city Addis Ababa. The University of Gondar Specialized Referral Hospital ART Clinic and Laboratory provides diagnostic and research services for over 5 million populations of Gondar town and the catchment Woreda inhabitants since 2003.

#### 2.1.1. Study Populations

In this study, a total of 200 people living with HIV who visited the ART clinic and laboratory seeking medical care and follow-up during the study period were included. While all HIV patients undergo the CrAg test participated, those who are chronically ill, unable to give consent, taking any antifungal medications, and diagnosed with cryptococcal infection with in the past couple of years did not take part in this study.

### 2.2. Data Collection

For every study participant who met the inclusion criteria, a structured questionnaire was used to collect sociodemographic (sex, age, education level, occupational, and marital status) and clinical data as well as presentations. Also, the patient's recorded medical history was reviewed for the presence of a previous infection of *cryptococcus*.

### 2.3. Specimen Collection and Processing

From each study participant, 5 ml of venous blood was collected using sterile test tubes separately by a trained laboratory technologist. Aliquots of specimens were used to CD4 cells count, HIV-1 RNA viral RNA, and hemoglobin determination (from whole blood) as well as CrAg LFA test for the detection of cryptococcal antigen (from plasma).

### 2.4. Laboratory Analysis

#### 2.4.1. Cryptococcal Antigen Testing

Following the collection of antecubital venous blood, plasma was obtained by standing the test tube, and the CrAg Lateral flow assay (LFA) test (Immy Diagnostics, Norman, Oklahoma, USA) was performed according to the manufacturer's instructions (http://www.immy.com/bluejuice/wp-content/uploads/2016/09/CR2003-CrAg-LFA-PI-US-1.pdf). *Summary of test principle*: The CrAg LFA test uses specimen wicking to capture gold-conjugated, anti-CrAg monoclonal antibodies and gold-conjugated control antibodies deposited on the test membrane. If CrAg is present in the specimen, then it binds to the gold-conjugated, anti-CrAg antibodies. The gold-labeled antibody-antigen complex continues to wick up the membrane where it will interact with the test line, which has immobilized anti-CrAg monoclonal antibodies. The gold-labeled antibody-antigen complex forms a Sandwich at the test line, causing a visible line to form. The procedure briefly was 40 *µ*L of LF Specimen diluent and 40 *µ*L of the specimen were added and mixed in a sterile Eppendorf tube; then, the white end of a CrAg LFA test strip was submerged into the test tube. Following 10 minutes of stay, the results were read. The test was interpreted as positive if both the test line and control line appeared, negative if only one line appeared on the control region, and invalid if there is a line on the test region but not on the control or there is no line development in the test and control regions at all.

#### 2.4.2. CD4 Th Cell Count

CD4 cell counting was done from a whole blood specimen using a FACSCalibur Immunecytometry analyzer (BD Biosciences, San José, USA) according to the manufacturer's instruction (https://www.bdbiosciences.com/documents/BD_FACSCalibur_instructions.pdf). *Summary of test principle*: while whole blood is mixed with fluorochrome-labeled antibodies in the reagents, the fluorescent-labeled antibodies bind specifically to white blood cell surface antigens; this is followed by addition of fixatives. During sample running, the cells pass through the laser light, which causes the labeled cells to fluoresce. This fluorescent light provides the information necessary for the instrument to identify and count the lymphocytes and CD4 T lymphocytes.

#### 2.4.3. HIV RNA Quantitative Detection

HIV RNA in EDTA anticoagulated plasma was quantitated by nucleic acid amplification technologies, Polymerase Chain Reaction (PCR), by The COBAS® AmpliPrep/COBAS® TaqMan® HIV-1 Test (Roche Diagnostics International Ltd, Rotkreuz Switzerland) (https://www.fda.gov/media/73824/download). *Summary of tests principle*: The COBAS® AmpliPrep/COBAS® TaqMan® HIV-1 Test is based on three major processes: initially, automated specimen preparation by a generic silica-based capture technique to isolate HIV-1 (utilizes 850 *µ*L of plasma), followed by RNA reverse transcription of the target RNA, with the thermostable recombinant enzyme Thermus species DNA Polymerase (Z05) and appropriate buffer, to generate complementary DNA (cDNA) and finally simultaneous PCR amplification of target cDNA and detection of cleaved dual-labeled oligonucleotide probe specific to the target. The amplification of HIV-1 RNA and HIV-1 QS RNA is measured independently at different wavelengths. This process is repeated for a designated number of cycles, with each cycle effectively increasing the emission intensity of the individual reporter dyes, permitting independent identification of HIV-1 RNA and HIV-1 QS RNA. The PCR cycle where a growth curve starts exponential growth is related to the amount of starting material at the beginning of the PCR. The COBAS® TaqMan® Analyzer automatically determines the HIV-1 RNA concentration for the specimens and controls. The HIV-1 RNA concentration is expressed in copies (cp)/mL. The conversion factor between HIV-1 RNA copies/mL and HIV-1 International Units (IU)/mL is 0.6 cp/IU, using the WHO 1st International Standard for HIV-1 RNA for Nucleic Acid-Based Techniques (NAT) (NIBSC 97/656).

### 2.5. Quality Assurance of Data and Laboratory Experiments

Sociodemographic and clinical data were collected using well designed, preassessed, and coded questionnaires by trained health professionals. Specimens were collected by sterile test tubes; reagents were stored in proper thermal storage (2–8 O°C) and checked for expiration and any of the damage before being used in the experiments. Negative and positive control reagents, as well as the batch printout for flags and comments, were checked for insurance of validity ahead of the test run. All laboratory analysis was performed according to the manufacturer's instruction and standard operational procedures were strictly implemented.

### 2.6. Statistical Analysis

Following the double checking of data for missed variables and values, it was entered into SPSS version 20.0 statistical software. Descriptive parameters were employed to present data findings. *p* values < 0.05 were considered statistically significant.

## 3. Results

### 3.1. Sociodemographic and Clinical Characteristics of Study Participants

A total of 200 HIV + patients having a CD4+ T-cell count of less than 100 cell/*μ*l have participated in this study. Of all the participants, the sex distributions were 105 (52.5%) males and 94 (47.5%) females with an age range of 15–65 (Mean 39.42 ± 9) years. The majority of 158 (79%) of participants were urban residents. The median CD4+ T-cell count and viral load measurement were 54 cells/*μ*l (Range, 2–97 cells/*μ*l) and 2.16 × 10^5^ RNA copies/ml (Range, 50–3.66 × 10^5^ RNA copies/ml), respectively. The mean hemoglobin (Hb) level of study subjects was 11.2 g/dL (SD: ±2.3). Most patients were classified as WHO clinical stage IV (58%) and were taking antiretroviral drugs (73.5%). Sixty-one percent of participants had a body mass index (BMI) greater than 18.5 kg/m^2^ (Table 1).

**Figure 1:**
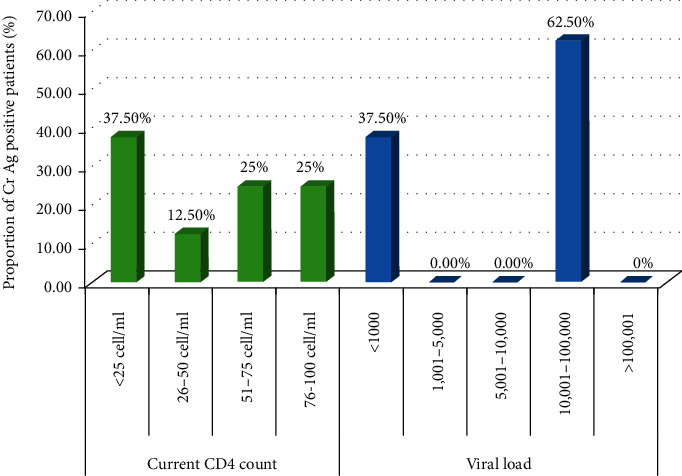
Cryptococcal antigenemia in relation to CD4 cells count and HIV viremia.

In this study, positive cryptococcal antigenemia was observed on 8 (4%) participants. Among these female patients, patients with lower CD4 count and those who had high viral load measurements were more likely to be diagnosed with positive cryptococcal antigenemia (*p* < 0.0001). Moreover, higher rates were observed in patients who are not on ART and had lower BMI (*p* < 0.0001). Hemoglobin level was not associated with a positive diagnosis of cryptococcal antigenemia (*p*=0.619). Moreover, most of Cr Ag positive patients had a CD4 count <25 cells/*μ*l and HIV viral load >10,000 copies/ml (Figure 1).

### 3.2. Clinical Aspects of Patients Screened for Cryptoccocal Antigenemia

At the time of *Cryptococcus's* diagnosis, 172 (86%) patients were presented with fever, 63.5% had a cough, 38% had diarrhea, and 25.5% had a headache. Detailed clinical signs and symptoms of these patients during the time of presentation are shown in Table 2. In the present study, a significant difference in the prevalence of cryptococcal antigenemia was observed in those patients with high-grade fever 6 (3.6%) and cough 6 (5%) than those without these clinical symptoms (*p* < 0.0001). Other infective conditions concurrently seen among the study participants include *tuberculosis* (15%), herpes zoster (10%), and oral candidiasis (12%). Lastly, we found a significant association between positive cryptococcal antigenemia and the presence of oral candidiasis (*p* < 0.0001).

## 4. Discussion

In addition to the presence of point-of-care tests having better diagnostic performance, the introduction of antifungal therapy and ART has reduced the occurrence of CM in western countries, but due to epidemicity of HIV, it has become one of the deadliest and commonest fungal infection among HIV infected patients in sub-Saharan Africa [25, 31–33]

In this hospital-based cross-sectional study, the prevalence of CrAg seropositivity was found to be 4% in HIV infected patients with a CD4+ T-cell count less than 100 cell/*μ*l and all the CrAg LFA test positive patients were linked to the University of Gondar Specialized Referral Hospital ART Clinic for proper medication and follow-up. Although the WHO recommends the CrAg test as a part of the routine screening among HIV patients, data on the burden of cryptococcal disease in Ethiopia are quite limited. Finding in the present study on the overall prevalence of positive serum CrAg has shown to be lower as compared with that of domestic studies including 10.2% in Adama [28], 8.3% in Gondar [34], 8.5% in Addis Ababa [26], while somewhat parallel with a recent finding from Mekelle which was 3.4% but higher than a 1.6% report from Adama [35, 36]. Apparently, the cryptococcal magnitude in Ethiopia has shown variability from time to time which might be an indicator in the consistency and efficacy of early ART initiation and adherence among HIV infected individuals as well as the introduction of antifungal therapy. Although a low prevalence of cryptococcal antigenemia does not necessarily mean a reduced prevalence of HIV in the community, the poor diagnostic facility in developing countries due to inconsistent supply of CrAg tests might be a probable explanation. Our finding also is comparable and/or slightly higher than findings from other African countries including South Africa (4.3%), Tanzania (3.7%), Namibia (3.3%), and Nigeria (1.4%) [37–40] but lower as compared to Nigeria (12.7%), Tanzania (7.1%), and India (33.3%) [41–43]. This variability in prevalence reflects the stringent case diagnosis and detection, an improved analytical competence, inconsistent HIV prevalence between nations, and the composition of study participants.

Sadly, in this study, a 24 years old HIV patient was diagnosed as CrAg positive and, the highest number of cryptococcosis patients was seen between the age group of 36–45 years (50%) followed by 26–35 years of age groups (37.5%). It has been hypothesized that interaction between cryptococcus and macrophages varies depending on gender and hormonal signatures leading to the fact that more males are prone to cryptococcosis than females [44]; however, our study revealed females accounted for 75% (6/8) of the total CrAg LFA tested positive patients.

Body mass index (BMI) is a decisive marker of nutritional status in patients with HIV infection as they develop substantial weight losses leading to immunocompromisation during the course of infection. In our study, 75% of cryptococcal infected HIV patients are underweight (<18.5 kg/m^2^), resulting in a statistically significant low BMI, and all patients with cryptococcal antigenemia are in WHO clinical stages III and IV.

According to our results, cryptococcal antigenemia is higher in patients with low CD4^+^ T-cell count and a third of CrAg testing positive HIV patients are below CD4^+^T-cell count of 25 cells/mm^3^ which supports the recommendation made by the WHO to undertake CrAg test and antifungal therapy for HIV infected patients particularly in those with CD4^+^T-cell count of <100 cells/mm^3^. Moreover, the current study demonstrated that cryptococcal antigenemia is associated with high viral load count which is in line with different studies done in different parts of the world [27, 45–47].

Interestingly, this study revealed that HIV patients with positive cryptococcal antigenemia have lower CD4+ T-cell levels and higher plasma HIV-1 viral loads, as compared to patients who were not positive for cryptococcosis. This might be due to immune-suppression in high Plasma HIV-1 viral load patients, which is commonly measured as an indicator of poor clinical response, and/or low CD4 lymphocyte count, an important surrogate marker of HIV disease progression that may predispose HIV infected patients to opportunistic infections.

Studies conducted around the globe showed the direct relationships of multiple clinical signs and symptoms in anticipating test positivity of serum cryptococcal antigenemia. In the present study, we have seen that clinical symptoms including fever, cough, and vomiting were significantly associated with cryptococcal antigenemia alike findings which were reported in a study conducted in Addis Ababa that enrolled febrile HIV-infected patients [48]. The release of microbial-derived urease in the lung promotes the accumulation of dendritic cells and nonprotective Th2 cells which alters the local immune response to the organism and cryptococcal polysaccharides which affects hemostasis, and in turn, produces clinical symptoms like cough and vomiting [49]; besides, progressive inflammation near the fungal-brain interface and the spinal cord results in a severe headache [50]. Studies from Oromia region of Ethiopia and Cambodia revealed a significant association between headache and serum cryptococcal antigen detection [28, 46] in contrast; this is not supported by our study as the highest percentage of our participants were on antifungal treatment and had a lumbar puncture which may result in the temporary relief of the symptoms.

Tuberculosis and candidiasis are among the most common concurrent diseases linked to the late stage of HIV infection. In our work, we noted that *Candida albicans* had a strong association with cryptococcal antigenemia positivity. The coexistence of TB, Candidiasis, and *Cryptococcus neoformans* may be explained by the devastating impact of HIV infection on CD4+ cell population's which has an important role in controlling such opportunistic infections [51]. In addition, non-HIV associated immune impairment induced by *M. tuberculosis* in coinfected patients may eventually lead to the occurrences of Candidiasis and Cryptococcus [52, 53].

Herpes zoster often occurs early in HIV infection and is not considered as AIDS-defining conditions; in contrast, cryptococcal meningitis is common in patients with advanced stages having very low CD4 counts [54]. Interestingly, ten percent of our study participants had a history of herpes zoster infection but did not show a statistically significant association with CrAg positivity.

As part of limitation, with this small number of sample size, we are unable to generate more statistics related findings, and generalization based on our finding is limited.

## 5. Conclusion

The clinical utility of LFA as a point-of-care test for the rapid diagnosis of cryptococcosis is ideal. Although the advent of ART, early diagnosis of cryptococcosis and application of antifungal interventions, HIV-induced cryptococcal antigenemia positivity in HIV infected individuals is still the countries' big challenge; thus, stringent follow-up and case management should be considered.

## Figures and Tables

**Table 1:** Sociodemographic and baseline characteristics of study participants.

Characteristics		Total (*n* = 200) (*n* = %)	Cr Ag Pos (*n* = 8); (*n* = %)	Cr Ag Neg (*n* = 192); (*n* = %)	*p* value
Sex	Male	105 (52.5)	2 (1.9)	103 (98.1)	0.001
	Female	95 (47.5)	6 (5.7)	88 (94.3)	
Age in years	15–25	8 (4)	1 (12.5)	7 (87.5)	0.437
	26–35	68 (34)	3 (4.4)	65 (95.6)	
	36–45	77 (38.5)	4 (5.4)	73 (94.6)	
	46–55	40 (20)	—	40 (100)	
	>56	7 (3.5)	—	7 (100)	
Residence	Urban	158 (79)	4 (2.51)	154 (97.9)	0.001
	Rural	42 (21)	4 (8.5)	38 (96.8)	
Marital status	Single	32 (16)	1 (3.2)	31 (96.8)	0.001
	Married	123 (61.5)	7 (6.0)	116 (94.0)	
	Divorced	38 (19)	—	38 (100)	
	Widowed	7 (3.5)	—	7 (100)	
CD4 count (*µ*l)	<25	30 (15)	3 (10)	27 (90)	0.003
	26–50	57 (28.5)	1 (1.8)	56 (98.2)	
	51–75	69 (34.5)	2 (2.9)	67 (97.1)	
	76–100	44 (22)	2 (4.5)	42 (95.5)	
On ART	Yes	147 (73.5)	5 (3.4)	142 (96.6)	0.001
	No	53 (26.5)	3 (5.7)	50 (94.3)	
BMI	<18.5 kg/m^2^	78 (39)	6 (8.3)	72 (91.7)	0.041
	>18.5 kg/m^2^	122 (61)	2 (1.6)	120 (98.4)	
WHO staging	Stage I	3 (1.5)	—	3 (100)	0.001
	Stage II	20 (10)	—	20 (100)	
	Stage III	61 (30.5)	3 (5.2)	58 (94.8)	
	Stage IV	116 (58)	5 (4.5)	111 (95.5)	
Hgb	<12 mg/dl	122 (61)	5 (4.2)	117 (95.8)	0.619
	>12 mg/dl	78 (39)	3 (4.0)	75 (96.0)	

**Table 2:** Presenting symptoms and concurrent opportunistic infection profile.

		Total (*n* = 200);(*n* = %)	Cr Ag Pos (*n* = 8);(*n* = %)	Cr Ag Neg (*n* = 192);(*n* = %)	*p* value
Presenting symptom	Headache	55 (27.5)	2 (3.8)	53 (96.2)	0.001
	Fever	172 (83)	6 (3.6)	166 (96.4)	
	Cough	127 (63.5)	6 (5)	121 (95)	
	Diarrhea	78 (39)	2 (2.7)	74 (97.3)	
	Vomiting	62 (31)	3 (5)	59 (95)	
	Nausea	178 (5)	—	17 (100)	
	Altered visual status	4 (2)	—	4 (100)	
	Neck stiffness	2 (1)	—	2 (100)	
	Dizziness	31 (15.5)	2 (0.7)	29 (99.3)	
Opportunistic infection	Herpes	20 (10)	0 (0.00)	20 (100)	0.001
	TB (pulmonary)	30 (15)	1 (0.3)	29 (99.7)	
	Chronic diarrhea	20 (10)	1 (5.3)	19 (94.7)	
	Oral/pharyngeal candidacies	24 (12)	4 (20)	20 (80)	

## Data Availability

The data used in this study are available in the manuscript; additional data will be accessed upon contact with the corresponding author.

## Abbreviations

AIDS: Acquired immunodeficiency syndrome
BMI: Body mass index
CM: Cryptococcal meningitis
Hb: Hemoglobin
LFA: Lateral flow assay
OI: Opportunistic infection
WHO: World health organization.

## References

[1] Pappas P. G., Alexander B. D., Andes D. R. (2010). Invasive fungal infections among organ transplant recipients: results of the transplant‐associated infection surveillance network (TRANSNET). *Clinical Infectious Diseases*.

[2] Anaissie D., Fishman J. A., Horn D. (2010). Epidemiology and outcome of invasive fungal infections in solid organ transplant recipients. *Transplant Infectious Disease*.

[3] Pfaller C., Fubin C., Jianghan C. (2012). Cryptococcosis in China (1985–2010): review of cases from Chinese database. *Mycopathologia*.

[4] Yalin D., Parris V. (2014). Cryptococcal meningitis: epidemiology and therapeutic options. *Clinical Epidemiology*.

[5] Rajasingham R., Smith R. M., Park B. J. (2017). Global burden of disease of HIV-associated cr yptococcal meningitis: an updated analysis. *The Lancet infectious diseases*.

[6] Elsegeiny W., Marr K. A., Williamson P. R. (2018). Immunology of cryptococcal infections: developing a rational approach to patient therapy. *Frontiers in Immunology*.

[7] Lemmer K., Naumann D., Raddatz B., Tintelnot K. (2004). Molecular typing ofCryptococcus neoformansby PCR fingerprinting, in comparison with serotyping and Fourier transform infrared-spectroscopy-based phenotyping. *Medical Mycology*.

[8] Cogliati M. (2013). Global molecular epidemiology of cryptococcus neoformans and cryptococcus gattii: an atlas of the molecular types. *Hindawi Publishing Corporation*.

[9] Johnston S. A., May R. C. (2010). The human fungal pathogen Cryptococcus neoformans escapes macrophages by a phagosome emptying mechanism that is inhibited by arp2/3 complex-mediated actin polymerisation. *PLoS Pathogology*.

[10] Levitz S. M. (2010). Innate recognition of fungal cell walls. *PLoS Pathogology*.

[11] Mansour M. K., Reedy J. L., Tam J. M., Vyas J. M. (2014). Macrophage-cryptococcus interactions: an update. *Current Fungal Infection Reports*.

[12] García-Rodas R., Zaragoza O. (2012). Catch me if you can: phagocytosis and killing avoidance by Cryptococcus neoformans. *FEMS Immunology and Medical Microbiology*.

[13] Walsh N. M., Wuthrich M., Wang H., Klein B., Hull C. M. (2017). Characterization of C-type lectins reveals an unexpectedly limited interaction between Cryptococcus neoformans spores and Dectin-1. *PLoS One*.

[14] Escandón P., Lizarazo J., Agudelo C. I., Casta˜neda E. (2018). Cryptococcosis in Colombia: compilation and analysis of data from laboratory-based surveillance. *Journal of Fungi*.

[15] Frola C., Guelfand L., Blugerman G. (2017). Prevalence of cryptococcal infection among advanced HIV patients in Argentina using lateral ﬂow immunoassay. *PLoS One*.

[16] McKenney J., Bauman S., Neary B. (2015). Prevalence, correlates, and outcomes of cryptococcal antigen positivity among patients with AIDS, United States, 1986–2012. *Clinical Infectious Diseases*.

[17] Doherty S., Shin G. Y., Wijewardana I. (2013). The prevalence of cryptococcal antigenemia in newly diagnosed HIV patients in a Southwest London cohort. *Journal of Infection*.

[18] Harrison E., Müller M. C., Ntamatungiro A. J. (2015). Cryptococcal antigenemia in immunocompromised human immunodeficiency virus patients in rural Tanzania: a preventable cause of early mortalityﬁciency virus patients in rural Tanzania: a preventable cause of early mortality. *Open Forum Infectious Diseases*.

[19] Battegay J. R., Lindsley M. D., Henchaison S., Poonwan N. (2012). High prevalence of cryptococcal infection among HIV-infected patients hospitalized with pneumonia in Thailand. *Clinical Infectious Diseases*.

[20] Jarvis J. N., Boulle A., Loyse A. (2009). ongoing burden of cryptococcal disease in Africa despite antiretroviral roll out. *AIDS*.

[21] Bicanic R., Smith R. M., Park B. J. (2017). Global burden of disease of HIV-associated cryptococcal meningitis: an updated analysis. *The Lancet Infectious Diseases*.

[22] Jarvis O., Poizat G., Zeller V. (2006). Long-term outcome of AIDS-associated cryptococcosis in the era of combination antiretroviral therapy. *AIDS*.

[23] Neuville S., Bor J., Nattey C. (2018). Persistent high burden of advanced HIV disease among patients seeking care in South Africa’s national HIV program: data from a nationwide laboratory cohort. *Clinical Infectious Diseases*.

[24] Glencross and COHERE Cohort Collaborations (2018). Global trends in CD4 CellCount at the start of antiretroviral therapy: collaborative study of treatment programs. *Clinical Infectious Diseases*.

[25] Assogoba K., Belo M., Wateba M. I. (2015). Neuromeningeal cryptococcosis in sub‐Saharan Africa: killer disease with sparse data. *Journal of Neurosciences in Rural Practice*.

[26] Bitew A., Hassen M., Getachew T., Fentaw S. (2016). Prevalence of crytpococcal infection in patients clinically diagnosed to have meningitis in Ethiopia. *Clinical Medicine Research*.

[27] Pongsai P., Atamasirikul K., Sungkanuparph S. (2010). The role of serum cryptococcal antigen screening for the early diagnosis of cryptococcosis in HIV-infected patients with different ranges of CD4 cell counts. *Journal of Infection*.

[28] Fekade T., Woldeamanuel Y., Asrat D., Ayana G., Boulware D. R. (2013). Comparison of cryptococcal antigenemia between antiretroviral naïve and antiretroviral experienced HIV positive patients at two hospitals in Ethiopia. *PLoS One*.

[29] Derbie A., Ayalew W., Mekonnen D., Alemu M., Mulugeta Y. (2018). Magnitude of cryptococcal antigenemia among HIV infected patients at a referral hospital, northwest Ethiopia. *Ethiopian Journal of Health Sciences*.

[30] WHO (2011). *Rapid Advice: Diagnosis, Prevention and Management of Cryptococcal Disease in HIV-Infected Adults, Adolescents and Children*.

[31] Boyer-Chammard T., Temfack E., Alanio A., Joseph J. N., Harrison T. S., Lortholary O. (2019). Recent advances in managing HIV-associated cryptococcal meningitis. *F1000Research*.

[32] Jarvis S., Ridolfo A., Fasan M. C. (2009). AIDS-associated cryptococcosis: a comparison of epidemiology, clinical features and outcome in the pre- and post-HAART eras. Experience of a single centre in Italy. *HIV Medicine*.

[33] GalimbertiMagni F., Mathoulin-Pélissier S., Fontanet A., Ronin O., Dupont B., Lortholary O. (2004). Epidemiology of HIV-associated cryptococcosis in France (1985–2001). *AIDS*.

[34] Seboxa T., Alemu S., Assefa A., Asefa A., Diro E. (2010). Cryptococcal meningitis in patients with acquired immunudeficiency syndrome in preHAART era at gondar college of medical Sciences hospital north-west Ethiopia. *Ethiopian Medical Journal*.

[35] Hailu K., Niguse S., Hagos K., Abdulkader M. (2019). Cryptococcal antigenemia and associated risk factors among ART‐naïve and ART‐experienced HIV‐infected peoples at selected health institutions of Mekelle, Northern Ethiopia. *Wiley Microbiology*.

[36] Reepalu A., Balcha T. T., Yitbarek T., Jarso G., Sturegard E., Bjorkman P. (2015). Screening for cryptococcal antigenemia using the lateral flow assay in antiretroviral therapy‐naïve HIV‐positive adults at an Ethiopian hospital clinic. *BMC Research Notes*.

[37] Longley N., Jarvis J. N., Meintjes G. (2016). Cryptococcal antigen screening in patients initiating ART in South Africa: a prospective cohort Study. *Clinical Infectious Diseases*.

[38] Govender E., Müller M. C., Ntamatungiro A. J. (2015). antigenemia in immunocompromised human immunodeficiency virus patients in rural Tanzania: a preventable cause of early mortality. *Open Forum Infectious Diseases*.

[39] Tanner S., Makumbi B., Purfield A. (2016). Estimated prevalence of Cryptococcus antigenemia (CrAg) among HIV‐infected adults with advanced immunosuppression in Namibia justifies routine screening and preemptive treatment. *PLoS One*.

[40] Chukwuanukwu R. C. (2019). Cryptococcus neoformans seropositivity and some haematological parameters in HIV seropositive subjects. *Journal of Infection and Public Health*.

[41] Oladele R. O., Akanmu A. S., Nwosu A. O., Ogunsola F. T., Richardson M. D., Denning D. W. (2016). Cryptococcal antigenemia in Nigerian patients with advanced human immunodeficiency virus: influence of antiretroviral therapy adherence. *Open Forum Infectious Diseases*.

[42] Magambo K. A., Kalluvya S. E., Kapoor S. W. (2014). Utility of urine and serum lateral flow assays to determine the prevalence and predictors of cryptococcal antigenemia in HIV-positive outpatients beginning antiretroviral therapy in Mwanza, Tanzania. *Journal of the International AIDS Society*.

[43] Lungran P., Vijaya D. A., Singh W. S. (2914). Cryptococcosis: its prevalence and clinical presentation among hiv positive and negative patients in rims, Manipur. *IOSR Journal of Dental and Medical Sciences (IOSR-JDMS)*.

[44] McClelland E. E., Hobbs L. M., Rivera J., Casadevall A., Potts W. K. (2013). The role of host gender in the pathogenesis of cryptococcus neoformans infections. *PLoS One*.

[45] French N., Gray K., Watera C. (2002). Cryptococcal infection in a cohort of HIV-1-infected Ugandan adults. *Aids*.

[46] Micol R., Lortholary O., Sar B. (2007). Prevalence, determinants of positivity, and clinical utility of cryptococcal antigenemia in Cambodian HIV-infected patients. *JAIDS Journal of Acquired Immune Deficiency Syndromes*.

[47] Geda N., Beyene T., Dabsu R., Mengist H. M. (2019). Prevalence of Cryptococcal Antigenemia and associated factors among HIV/AIDS patients on second-line antiretroviral therapy at two hospitals in Western Oromia, Ethiopia. *PloS One*.

[48] Alemu A. S., Kempker R. R., Tenna A. (2013). High prevalence of cryptococcalantigenemia among HIV-infected patients receiving antiretroviral therapy in Ethiopia. *Plos One*.

[49] Casadevall A., Coelho C., Alanio A. (2018). Mechanisms of Cryptococcus neoformans-mediated host damage. *Frontiers in Immunology*.

[50] Lee S. C., Kress Y., Zhao M.-L., Dickson D. W., Casadevall A. (1995). Cryptococcus neoformans survive and replicate in spacious phagosomes in human microglia. *Lab Invest*.

[51] de Repentigny L., Lewandowski D., Jolicoeur P. (2004). Immunopathogenesis of oropharyngeal candidiasis in human immunodeficiency virus infection. *Clinical Microbiology Reviews*.

[52] Chen X. H., Gao Y. C., Zhang Y., Tang Z. H., Yu Y. S., Zang G. Q. (2015). Tuberculosis infection might increase the risk of invasive candidiasis in an immunocompetent patient. *Revista do Instituto de Medicina Tropical de São Paulo*.

[53] Van Tongeren L., Shaipanich T., Fleetham J. A. (2011). Coinfection withCryptococcus GattiiandMycobacterium tuberculosisin an otherwise healthy 18-year-old woman. *Canadian Respiratory Journal*.

[54] WHO (2011). *HIV-Related Opportunistic Diseases*.

